# A case of methylprednisolone treatment for metronidazole-induced encephalopathy

**DOI:** 10.1186/s12883-019-1278-6

**Published:** 2019-03-30

**Authors:** Li Li, Xiaogang Tang, Wenlei Li, Seng Liang, Qing Zhu, Minghua Wu

**Affiliations:** 1 Department of neurology, Jiangsu Province Hospital of Chinese Medicine, 155 Hanzhong, Nanjing, 210029 Jiangsu Province China; 2 The first clinical medical college, Nanjing University of Chinese Medicine, Nanjing, 210023 Jiangsu Province China

**Keywords:** Metronidazole-induced encephalopathy, Cerebellar dentate nuclei, Methylprednisolone

## Abstract

**Background:**

Metronidazole, a common antimicrobial agent, can induce encephalopathy in rare cases. After discontinuing metronidazole, most patients show clinical improvement. However, in the face of deteriorating conditions, there have done not to have reports of effective drug treatment.

**Case presentation:**

A 57-year-old man was admitted to our hospital due to dysarthria and ataxic gait after taking metronidazole at the dose of about 32 g for 20 days. Neurological examination showed that his upward and outward movements of bilateral eyeballs were limited, and horizontal and vertical nystagmus were noted. The brain magnetic resonance imaging showed hyper-intensities in the bilateral cerebellar dentate nuclei, medulla oblongata, midbrain and red nuclei in T2W and FLAIR images. However, the patient’s clinical symptoms worsened after drug cessation. High-dose intravenous methylprednisolone pulse therapy was applied, and this led to a drastic improvement of his symptoms and signs.

**Conclusions:**

In our case, we suggest that early methylprednisolone intervention can prevent the progression of metronidazole-induced encephalopathy and accelerate neurological recovery. We infer that the progression of encephalopathy is related to the delayed toxicity caused by high dose or concentration of metronidazole.

## Background

Metronidazole is a derivative of nitroimidazoles and is widely used as an antimicrobial agent to treat amebiasis, *Helicobacter pylori* and other anaerobic infections. In rare cases, metronidazole may produce neurotoxicity. Metronidazole-induced encephalopathy (MIE) is an extremely rare disease caused by fatal adverse reactions to metronidazole. Most MIE cases are reversible following discontinuation, but some are fatal [1, 2]. So far, there have been no reports on effective treatment to address the deteriorating condition after metronidazole intake. Here, we report for the first time, a 57-year-old man with MIE whose neurological symptoms were successfully treated with methylprednisolone with complete remission of symptoms.

## Case presentation

**Fig. 1:**
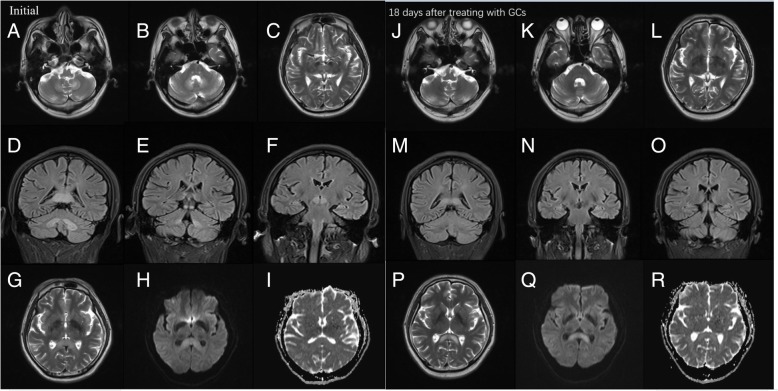
MRI findings. Scanning was performed by using 3.0 T MR imaging systems (Siemens). Initial MRI findings.T2W MRI images show hyperintensities in bilateral cerebellar dentate nuclei (**a**), medulla oblongata (**a**), midbrain (**b**) and red nuclei (**c**). Coronal FLAIR images show hyperintensity in bilateral dentate nuclei (**d**), splenium of the corpus callosum (**d**), gray matter near the midbrain ducts (**e**) and red nuclei (**f**). Moreover, T2W image shows slight hyperintensity in splenium of corpus callosum (**g**), but DWI and ADC images only show diffusion restriction in the corresponding areas of corpus callosum (**h**, **i**). Follow-up MRI imaging was conducted 18 days after treating with GCs (22 days after discontinuation of metronidazole). T2W images (**j**-**l**) and coronal FLAIR images (**m**-**o**) show complete resolution of the previously noted signal changes. However T2W image shows higher hyperintensity in splenium of corpus callosum in axial (**p**). DWI and ADC images show diffusion restriction and a high ADC respectively in the corresponding areas of corpus callosum (**q**, **r**)

A 57-year-old man was admitted to our hospital after displaying symptoms of dysarthria and ataxic gait for 2 days. The patient was prescribed oral metronidazole (400 mg four times a day) and levofloxacin (200 mg twice a day) for 20 days due to nasosinusitis. No other drugs were prescribed or used by the patient in the 20 days before admission to our hospital. He had hypertension, type 2 diabetes, and chronic nephritis, but no history of alcohol abuse. He did not take any other medications, such as chemotherapy or antiepileptic drugs. During diagnosis, neurological examination showed that the upward and outward movements of bilateral eyeballs were limited, and horizontal and vertical nystagmus were observed. The muscular tension of his four limbs was weak, tendon reflex (+), meningeal irritation sign (−), bilateral Babinski sign (+), the deep and superficial sensibility of the limbs were normal, and bilateral finger-nose test and heel-knee-tibia test could not be completed. Laboratory findings showed that Aspartate aminotransferase (AST) 58 U/L, serum alanine aminotransferase (ALT) was 61 U/L, total protein was 62.86 g/L, γ-glutamyltransferase (GGT) was 107 U/L, uric acid was 157.5 umol/L, glutamic acid dehydrogenase was 8 g/L, sugar level was 6.66 mmol/L, and potassium level was 3.08 mmol/L. Lumbar puncture revealed that the level of protein in cerebrospinal fluid (CSF) was increased to 893 mg/L, but other CSF test results were normal. The brain magnetic resonance imaging (MRI), performed 20 days after initiation of metronidazole, showed hyper-intensities in the bilateral cerebellar dentate nuclei, medulla oblongata, midbrain, red nuclei and corpus callosum on T2 FLAIR images. In addition, hyperintense lesions were observed in the corresponding areas of the corpus callosum on DWI images. In the present case, there were no other white matter abnormalities on MRI. (Fig. 1a-j).

The offending drug was discontinued immediately, but on the 3rd day of in-hospital stay, he was unable to stand or walk. Also, the patient showed signs of confusion. We administered high-dose of intravenous methylprednisolone pulse therapy (500 mg/day). Meanwhile, 100 mg thiamine was given by muscular injection and 1 mg Vitamin B12 was given by intravenous injection. On the 4th day of his hospital stay and on the 2nd day after receiving glucocorticoids treatment, his dysarthria improved significantly and he was able to walk, albeit with some balance issues. On the 5th day, the dose of glucocorticoids (GCs) was progressively reduced. Half a month later, the patient’s symptoms resolved completely. Follow-up MRI examination showed that almost all lesions disappeared but the splenium of corpus callosum residual had lesions 18 days after initiation of GCs. (Fig. 1k-t).

## Discussion

We have described a rare case of MIE that was aggravated after drug withdrawal. In the face of deteriorating patient’s symptoms, we initiated an early treatment of methylprednisolone, which was highly effective and prevented fatal outcomes.

CNS symptoms of metronidazole-induced encephalopathy (MIE) include ataxia related to cerebellar dysfunction, dysarthria, altered mental status, seizure, and coma [2]. Up to 75% of such patients have cerebellar dysfunction, mostly manifested as ataxia and dysarthria [3]. Recent literature indicates that the neurotoxicity caused by metronidazole does not vary with the dose, duration of administration, or route of administration (oral or intravenous) [4]. The total dose and duration of metronidazole that causes MIE have been reported as 20 g - 120 g and 1–12 weeks, respectively. In our case, the total dose of metronidazole taken orally by the patient was about 32 g, and the duration was 20 days. Although similar cases of intoxication have been reported in self-medicated long-term drug use [5], this patient encountered this problem within a short time after taking metronidazole. According to a previous literature review, the time to symptoms remission after metronidazole discontinuation varies from few days to several weeks [6], and median time to recovery of central nervous function is 23.3 days [7]. It can be deduced that the time to improvement of neurological deficits depends on various factors including duration and severity of clinical features as well as other concomitant diseases. His status did not show a rapid improvement after discontinuation of metronidazole, which may be due to a delayed effect caused by drug accumulation [8].

The most characteristic imaging feature of MIE is the symmetric hyperintensity of the cerebellar dentate nuclei in T2W and FLAIR images [9], which may be accompanied with bilateral symmetric hyperintensity within the mesocerebrum, dorsal pontine and the splenium of the corpus callosum in T2W images. Other rarely involved locations are the cerebral white matter and basal ganglia. In our case, MRI showed bilateral symmetric hyperintensity of cerebellar dentate nuclei, medulla, red nuclei and the splenium of corpus callosum in T2W or FLAIR images. A follow-up MRI examination at 18 days after GCs initiation indicated that the foci of cerebellar dentate nuclei had disappeared completely, but residual foci of the splenium of corpus callosum hyperintensity were found in T2W, FLAIR and DWI images. The mechanism by which MIE causes brain damage remains unclear. According to the findings of symmetry reversible imaging in most cases (including our case), Ahmed et al. first proposed that neurotoxicity caused by “axonal swelling with increased water content” may lead to the pathogenesis of brain damage [10]. Later on, following reports on medication-induced Acute Toxic Leukoencephalopathy, it was gradually realized that metronidazole generates superoxide radicals and hydrogen peroxide from its ingredients, eventually forming intramyelinic edema and myelin vacuolation [11, 12]. Hence, this cytotoxic effect causes brain damage and has been corroborated by pathological examinations [13].

In most cases, the brain damage induced by metronidazole is reversible. It has been shown that symptoms normalizes within few days after drug withdrawal [14]. However, in our case, 3 days after drug cessation, the patient’s condition deteriorated. It has previously been observed that metronidazole toxicity can lead to fatal consequences. To prevent complications, we administered methylprednisolone treatment for the first time. Methylprednisolone is a steroid that produces non-specific immunosuppressive effects in the central nervous system. In so doing, it alleviates tissue inflammation and cellular edema thereby promoting microcirculatory perfusion to enhance cerebral local blood flow. The antioxidative effect of methylprednisolone has a primary protective effect on neurons and improves lysosomal stability [15, 16]. In view of the fact that there is no effective drug to prevent complications after stopping metronidazole, we cautiously selected methylprednisolone based on the pathogenesis of the disease and the characteristics of this steroid, mainly its ability to eliminate edema and its antioxidant effects, although there is no case report of using steroid for this type of case. Therefore, we provided supportive care and strict safety monitoring. Surprisingly, the therapy was found to be effective. This indicates that MIE should be identified early, and the drug should be stopped as soon as possible and other treatment options should be explored to prevent fatal outcomes. Based on past experiences, we did not think it is the role of thiamine [2], considering 100 mg thiamine cannot promptly improve his dysarthria. The mechanism by which methylprednisolone resolved the patient’s condition is not clear but we speculate that it is linked to its properties of inhibiting superoxide radicals, reducing axonal edema caused by hypoxic-ischemic injury [17], and increasing the expression of gamma-aminobutyric acid (GABA) [18]. Thus, it may be a new treatment for patients whose symptoms gradually deteriorate after drug withdrawal. Early methylprednisolone intervention in MIE patients may prevent complications and accelerate neurological recovery.

## Conclusion

In conclusion, we have presented a case of MIE that worsened after drug cessation. We consider that the early methylprednisolone intervention implementation can prevent progression of metronidazole-induced encephalopathy and reduce neurological recovery time. We consider that the progression of MIE is related to the delayed toxicity or persistent drug effect in blood or brain. Further research should be undertaken to investigate the mechanisms of GCs’ neuroprotection in MIE.
